# Response in Behalf of the Graduates

**Published:** 1859-03

**Authors:** B. B. Ward

**Affiliations:** Mobile, Ala.


### RESPONSE IN BEHALF OF THE GRADUATES.

BY B. B. WARD, D. D. S., OF MOBILE, ALA.

GENTLEMEN:—To the very able valedictory, it would be difficult to reply; and before so many men of science, to say the least, it is embarrassing for one so young in the dental profession.

We appear before you to-night with the lonely word "good-bye," upon our tongues. It is, indeed, a lonely word, frequently causing a tear to start in the eye, and the lips of the strong man to quiver.

Your excellent address bids us God-speed in our profession; and, as young men, looking into the unseen future, your wise counsels are well fitted to guide us through time. I trust that each of us will bind them upon our heads as an ornament, and as a chain of gold about our necks.

The breaking up of a happy household is a scene of no ordinary character; and such a one as this to-night, brings it in bright review. During the past four months, no word has arisen to mar the perfect harmony that has prevailed. Looking into the past, how ably have you discharged the duties that have devolved upon you! At no moment has your energy flagged,—at no hour have your chairs been vacated,—not an item has been omitted that would tend to our advancement.

At the beginning of our session, like an uneducated man looking into a vast piece of machinery, contemplating confusion and disorder, or like an unlettered peasant gazing on the starry heavens, to him a mystery, but to the astronomer's eye all is clear, so we, then looked upon the science of dental surgery with confused minds. But, guided by your light and knowledge, we have seen clearly the end from the beginning.

We might trace the history of dental surgery back to the days of the Pharaohs, when the hot iron, or the boiling oil, was used for the relief of pain; or the palmy days of the Roman Empire, when, like a bud upon the parent stem, she began to grow, liable at any moment to be blasted by the frost of a jeering public. But she bloomed and blossomed, and grew up, the mighty offspring of the parent stem. And how does she stand to-day but like a mighty oak, spreading out its branches and thousands of our fellow creatures seeking relief beneath her shade?

To whom then are we indebted for this noble science, but to such men as you? Men who have gone before you, whose places you now occupy. Men who have drunk deep and tasted of the pierian spring of science. To you and such as you are we indebted for high attainments in our profession. Unlike the selfish man, you have not hid them in a napkin, but have unfolded them to the world, that your fellow beings might be benefited. To relieve the suffering, to instruct the young, has been your aim. What can we render you for such services? What can we offer you in remuneration? Like the Caffre chief released from his fetters, *nothing* but a grateful heart.

When God dipped his finger into the chaos, and dispelled the gloom of the dark ages,—when the sun of Christianity shed forth his light,—when knowledge was increased,—then did dental surgery spring into existence. A few men of medicine turned aside from their legitimate profession, and with the rude instruments of the day, began extraction. But the formative process, employed by nature's God, in the growth of the teeth, was to them a mystery. Without the compass of literature to guide them, with no physiological charts, they lowered their frail boat upon the waves of trouble, and amid the perverse winds of persecution, undaunted and brave, they pushed on to the goal, and laid the foundation of our noble institution. Others soon enlisted beneath their banners. Hunter and Fox, of England, Cuvier and Serres, of France;—but the true history of the development of the teeth was reserved for such men as Retzius and Owen, Arnold and Goodsir. Nor has your energy failed. Following in the footsteps of your predecessors, you have added vastly to this science, built up, in this your city, a noble edifice for the advancement of the profession,—adding much to her literary fame. Self sacrificing in your efforts, you give your time and labor for the advantge of the young; and, ever striving to alleviate the sufferings of humanity, high is the position that you occupy, and of vast importance are your labors to the world. Go on, we say, and God speed you! Weary not in your laborious task; and we will follow in your footsteps.

FELLOW GRADUATES:—How shall I address you? For the past four months we have gone hand and hand in our studies—side by side with the duties that have devolved upon us. How we have discharged these duties is known to us now; for we bear in our hands the evidences of our instructors' approval. Shall we ever bring a stain upon the institution that has given us position in the medical and scien tific world? or dishonor upon the names of those who have labored for our advancement? I trust not. But in whatever community we may locate, let us

> "Pitch our behavior low, our projects high,
> So shall we humble and magnanimous be;
> Link not in spirit,—who aimeth at the sky
> Shoots higher much than he that means a tree."

We separate some for the north, others for the far south, —it may be for years,—or it may be for ever,—but the reminiscences of the past months should never be forgotten. Write the lessons of instruction upon the tablets of your hearts, with a pen of iron, that they may never be defaced. Let us carry home the principles given to us in the Ohio College. Let us aim to have an irreproachable character, an honorable name. Following in the footsteps of our professors, we may soon attain a position among our fellow creatures, that will be respected and revered.

To those students we leave behind, let me say a word. Pursue your studies within these walls. Be guided by those who have guided us. Respect them for their knowledge; honor them for their ages, and a year hence may see you occupying the position we do to-night. And when death steals o'er us, though dead we may yet speak. In passing through life we may strew our pathway with the richest flowers, or leave indellible impressions of our presence in time. Hallowed will then be our names and green our memories. Then, up! up!! as if borne upon the wings of the wind, far above suns and systems. Up! until the watchers upon the battlements of heaven hail us with a joyous shout, "well done faithful servants."

There, gentlemen, we leave you, with the best wishes of your graduating class of '59.

